# Short Versus Long Antibiotic Therapy and Risk of Recurrence of Acute Cholangitis Due to Malignant Biliary Strictures

**DOI:** 10.3390/jcm12216716

**Published:** 2023-10-24

**Authors:** Philip G. Ferstl, Katharina Bremer, Natalie Filmann, Volkhard A. J. Kempf, Michael Hogardt, Olivier Ballo, Fabian Finkelmeier, Jonel Trebicka, Stefan Zeuzem, Jörg Bojunga, Mireen Friedrich-Rust, Dirk Walter

**Affiliations:** 1 Goethe University Frankfurt, University Hospital, Medical Clinic 1, 60590 Frankfurt, Germanyfinkelmeier@med.uni-frankfurt.de (F.F.); jonel.trebicka@ukmuenster.de (J.T.); stefan.zeuzem@kgu.de (S.Z.); joerg.bojunga@kgu.de (J.B.); mireen.friedrich-rust@kgu.de (M.F.-R.); d.walter@med.uni-frankfurt.de (D.W.); 2 Goethe University Frankfurt, Institute of Biostatistics and Mathematical Modeling, 60590 Frankfurt, Germany; filmann@med.uni-frankfurt.de; 3 Goethe University Frankfurt, University Hospital, Institute for Medical Microbiology and Infection Control, University Center of Competence for Infection Control of the State of Hesse, 60590 Frankfurt, Germany; volkhard.kempf@kgu.de (V.A.J.K.); michael.hogardt@kgu.de (M.H.); 4 Goethe University Frankfurt, University Hospital, Medical Clinic 2, 60590 Frankfurt, Germany; 5 Department of Internal Medicine B, University of Münster, 48149 Münster, Germany; 6 European Foundation for the Study of Chronic Liver Failure, 08021 Barcelona, Spain

**Keywords:** cancer-associated cholangitis, multidrug resistance, metal stent, antibiotic duration, recurring cholangitis

## Abstract

Malignancies can cause severe stenosis of the biliary tract and therefore predispose a patient to bacterial cholangitis. Upon endoscopic drainage, antibiotic therapy (AT) is performed according to individual clinical judgement, as the optimal duration of AT is unclear to date, especially in the case of multidrug-resistant organisms (MDROs). In a case-based retrospective study, patients with malignant biliary strictures and acute cholangitis were included upon endoscopic retrograde cholangiography (ERC). The outcome of cases treated with short AT (≤6 days) was compared to that of long AT (≥7 days). Recurrent cholangitis (RC) before scheduled stent exchange was the primary end point. In total, 124 patients were included, with 183 cases of proven cholangitis in total. The overall median duration of AT was 7 days (range 1–20), with 74 cases (40%) receiving short AT and 109 (60%) receiving long AT. Short AT was not an independent risk factor for RC (HR = 0.66, *p* > 0.2), while colonization with MDROs was associated with a higher risk of RC (HR = 2.21, *p* = 0.005). Placement of a metal stent was associated with minor risk of RC (HR = 0.4, *p* = 0.038). In conclusion, short AT is possible in selected patients with non-severe cholangitis and malignant biliary strictures. Scheduled screening for MDROs is recommended and placement of a metal stent should be performed if possible.

## 1. Introduction

Bacterial cholangitis is a common, potentially life-threatening complication in patients with a malignant biliary stricture. Cancer-associated cholangitis (CAC) will result from impaired flow of bile into the intestine, leading to stasis and infection of the biliary tract. Pancreatic carcinoma and distal cholangiocarcinoma are the main reasons for stenosis at the distal common bile duct, while proximal stenosis is mostly caused by perihilar cholangiocarcinoma. Intrahepatic stenoses of the biliary tree are most frequently due to hepatocellular carcinoma, intrahepatic cholangiocarcinoma, lymph node metastasis or liver metastasis [1].

Regardless of the location of the stenosis, in case of infection, a therapy to restore the bile flow is urgently warranted. Endoscopic retrograde cholangiography (ERC), most often including stent insertion, is usually the primary treatment of choice. Furthermore, adequate antibiotic treatment (AT) is a therapeutic cornerstone. Selection of the antibiotic regimen depends on the expected range of pathogens, i.e., mostly *Enterobacteriaceae* [2,3]. AT should be as long as necessary, and as short as possible, but no concise recommendations regarding length in days have been made in patients with CAC to date. The general recommendation for the duration of AT for cholangitis according to the Tokyo guidelines 2018 (TG18) is 4–7 days once the source of infection is controlled [3]. Extension to 14 days should be performed in case of Gram-positive bacteremia [3]. However, data leading to the recommendations in the TG18 are mainly derived from small patient cohorts and mostly benign etiologies of biliary strictures. Drawing conclusions on the management of patients with CAC is therefore hindered. Moreover, data on risk factors for recurrent cholangitis in this population are lacking to date.

The high importance of an optimal duration of AT is underlined by the steady incline in multidrug-resistant organisms (MDROs) in patients with malignancies and its association with impaired survival [4,5,6]. Therefore, AT and hospitalization should only be as short as necessary in order to avoid further selection of MDROs in this vulnerable population. On the other hand, due to often incomplete biliary drainage, one must be careful not to over-shorten AT.

Against this background, the aim of this study was to assess the potential impact of the duration of AT on the recurrence of cholangitis in patients with CAC.

## 2. Materials and Methods

### 2.1. Data Curation

**Figure 1:**
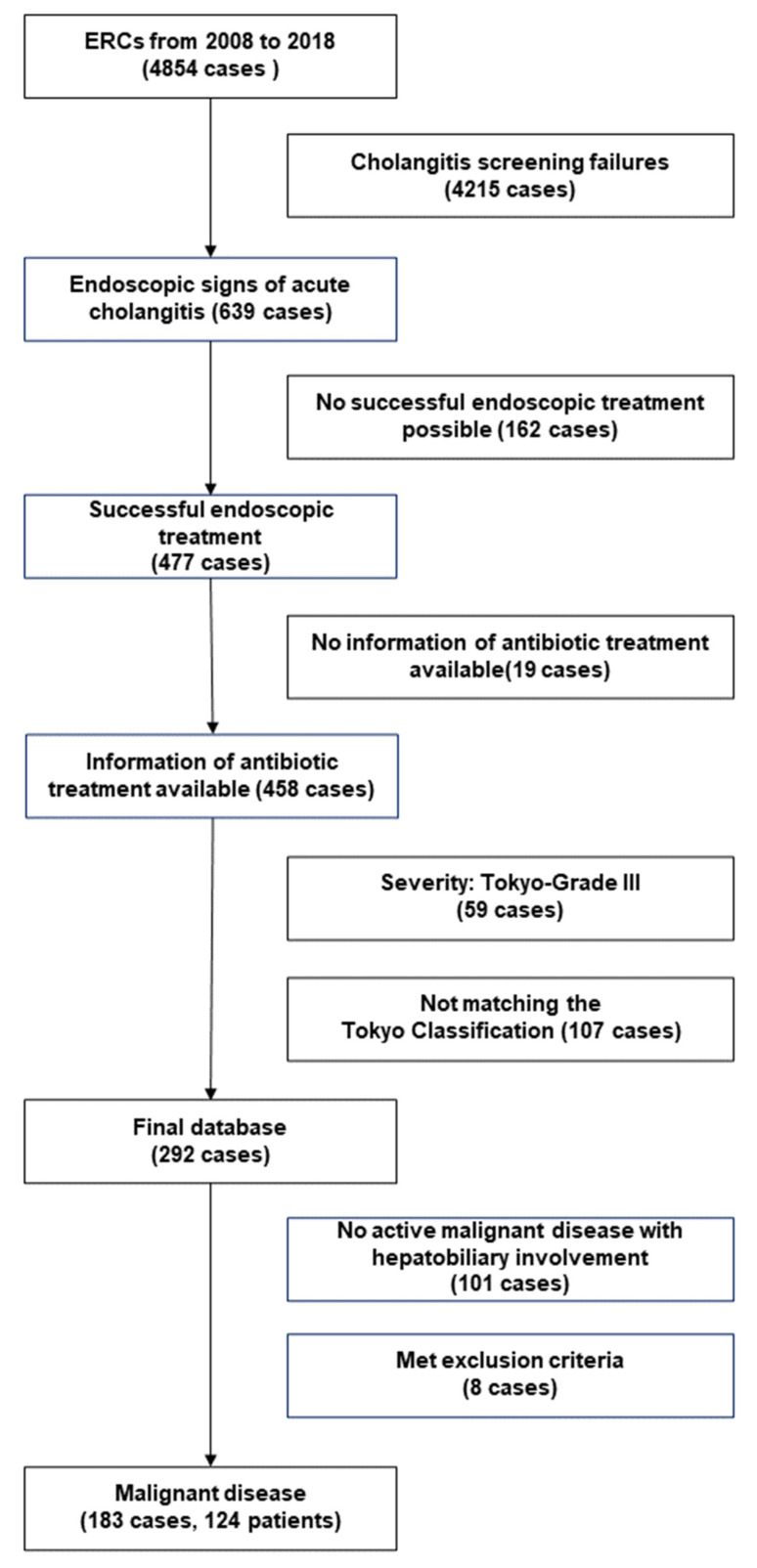
Consort diagram of the enrollment algorithm.

In this retrospective study, all patients admitted to a tertiary German liver unit between 2008 and 2019 for inpatient treatment of cholangitis with ERC were admitted. Eligible patients were screened for in an endoscopic database containing all ERC cases between 2008 and 2018, as described in our previous study, and followed up for at least one year (Figure 1) [7]. Screening key words were any endoscopic findings indicating pus, turbid bile, fluoroscopic evidence of cholestasis and/or other endoscopic findings backing the diagnosis of cholangitis within all ERC reports. Then, cases not matching laboratory criteria in the TG18 for cholestasis and systemic inflammation were removed [2]. Patient charts were then evaluated including biometrical, clinical and microbiological data as well as antibiotic therapies. Exclusion criteria were transcutaneous biliary drainage, permanent biliary focus such as permanent leakage or abscedation, cholangitis grade 3 and additional focus of infection with longer treatment requirements than cholangitis itself. The database was anonymized, and ethical approval was obtained prior to study initiation (file number 74/19).

### 2.2. Case Definition and Treatment Rationale

In the present study, patients were included if they matched each of the following requirements: (A) currently active malignant disease inflicting stenosis of the CBD and/or subbranches of the biliary tree, (B) age of at least 18 years and (C) cholangitis of grade 1 or 2, according to the TG18 criteria [2,3,7]. Cases with grade 3 cholangitis were excluded since these patients often require longer AT. The duration of AT was assessed starting from the day of ERC, and cases were stratified into two groups depending on the length of AT of less than (short AT) or more than six days (long AT). A six-day cut-off was chosen in line with data from the literature as well as currently ongoing clinical trials [7,8]. The end point was the occurrence of RC, which was defined as any episode of cholangitis requiring ERC after a previous episode of cholangitis.

In cholangitis, causative pathogens may be expected to be mostly due to *Enterobacterales* or non-fermenting bacteria such as *Pseudomonas aeruginosa* or *Acinetobacter baumannii,* but may also be caused by *Enterococcus* spp. Regarding the choice of antibiotic substances, 3rd/4th generation cephalosporins were administered if no MDRO colonization was known or suspected at diagnosis of cholangitis. Piperacillin/tazobactam or fluoroquinolones were used in cases with previous evidence of extended-spectrum ß-lactamase (ESBL)-expressing bacteria, and carbapenems were the substance of choice, e.g., in the case of MDRGN with susceptibility towards carbapenems (see below). MDROs were therefore defined as either one of the following, as described before: multidrug-resistant Gram-negative bacteria (MDRGN) or vancomycin-resistant enterococci (VRE) [8]. In particular, MDRGN were defined as *Enterobacterales* with an extended spectrum beta-lactamase phenotype as well as *Pseudomonas aeruginosa*, *Enterobacterales* and *Acinetobacter baumannii* resistant to any 3rd/4th generation cephalosporins, Piperacillin and fluoroquinolones [9]. MDROs were screened for and detected as described earlier [10,11]. Cases were only considered MDRO-positive if detection had been made prior to ERC, meaning that only primary MDRO carriage was ruled in.

### 2.3. Statistical Analysis

Categorical variables were displayed as frequencies and percentages, and continuous variables as medians with ranges or—as appropriate—as means with interquartile ranges. For comparisons at baseline, the Wilcoxon–Mann–Whitney U-test and the Chi-Square test were used. Independent risk factors for RC were determined in a two-step approach using A) univariate Cox-regression where significant parameters were exclusively assessed with B) multivariate Cox-regression. The results are displayed as hazard ratios (HR) and 95%-confidence intervals (95%-CI) are depicted for statistical significance. Importantly, survival was not an end point in the current study. All tests were two-sided. *p*-values ≤ 0.05 were considered significant. R version 4.0.4 software was used for all statistical analyses (R Core Team (2021), R Foundation for Statistical Computing, Vienna, Austria).

## 3. Results

### 3.1. Study Population

In the present study, 124 individuals with malignant CBD and/or subbranch stenosis were included. Among these, 183 cases of acute cholangitis according to the (TG18) criteria were identified (Figure 1).

Thereof, 181/183 (99%) were clinically significant and required AT. The baseline characteristics of the 124 patients are shown in Table 1.

### 3.2. Treatment of Cholangitis

On average, patients underwent ERC 35.6 h after hospital admission, and stenting was performed in 176 (96%) instances (Table 2).

Endoscopic drainage was considered complete in 134 (73%) cases. In all patients, antibiotics were administered in a periprocedural manner (i.e., before, during or immediately after ERC) and AT was initiated at least one day prior to ERC in 172 (94%) cases. The overall median duration of AT was 7 (range 1–20) days in the entire cohort. Short AT was performed in 74 (40%) cases with a median duration of 5 days (range 1–6), and LAT was performed in 109 cases (60%), with a median AT of 9 days (range 7–20). Notably, baseline total bilirubin, international normalized ratio (INR) and aspartate-aminotransferase were significantly higher in patients with LATs (Table 2). Carbapenems (*n* = 31, 17%), piperacillin/tazobactam (*n* = 36, 20%), cephalosporines (*n* = 76, 42%) and/or fluoroquinolones (*n* = 43, 23%) were classes of initially administered AT. Importantly, the duration of AT did not differ between these types of antibiotics. In two and three cases, respectively, a fluoroquinolone was combined with piperacillin/tazobactam or a cephalosporine. Adjustment of AT was necessary in 23 (13%) cases. AT could be switched to oral administration in 124 cases (68%) to facilitate earlier discharge.

### 3.3. Microbiological Testing

Blood cultures were taken in 64 cases (35%), whereof 40/64 cultures (62%) were sterile, 7/64 (11%) were positive for Gram-negative bacteria, 15/64 (23%) were Gram-positive, and 2 (3%) were polymicrobial. Colonization with MDROs was present in 51 (28%) patients with cholangitis and was comparably distributed among the short and long AT groups (Table 2). MDRGN were the predominant pathogen (40 cases, 22%). One strain of carbapenem-resistant *K. pneumoniae* was detected, and 37 cases (20%) were positive for VRE (Table S1).

### 3.4. Recurrent Cholangitis

**Figure 2:**
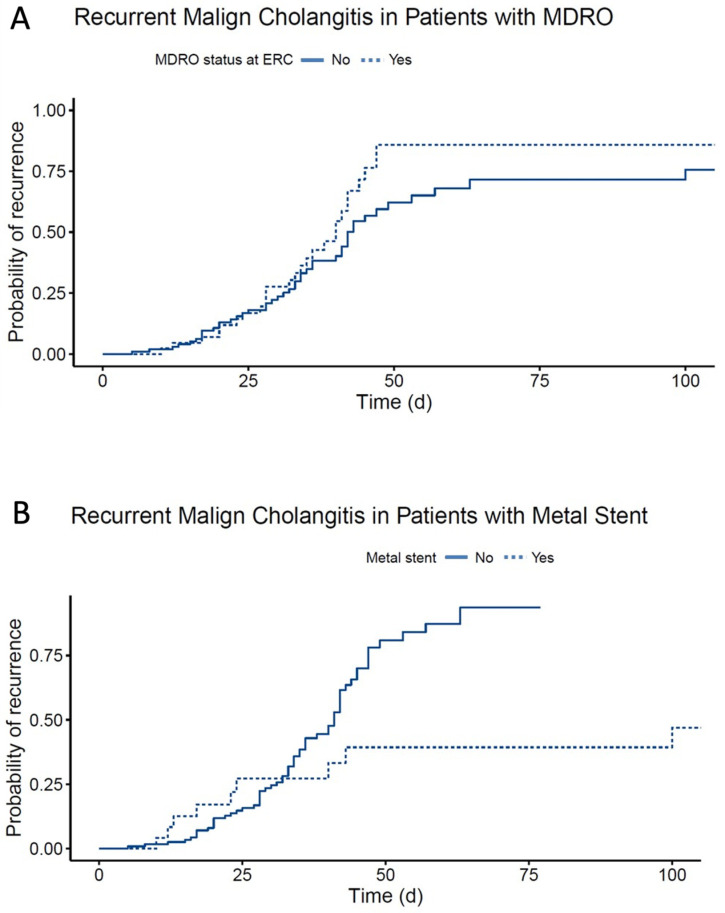
Cumulative incidence plots of patients developing recurrent cholangitis upon endoscopic drainage of malign cholangitis via endoscopic retrograde cholangiography. (**A**) Patients with and without multidrug-resistant organisms. (**B**) Patients with and without a metal stent.

In total, RC was observed in 73/183 cases (40%), of whom 37/73 (50%) were in the short AT and 36/109 (33%) in the long AT group. The median AT was 6 days (range 1–16) in cases developing RC as opposed to 7 days (1–20) in cases without subsequent RC. Furthermore, only 7/24 (29%) of the cases with positive blood cultures developed RC, compared to 14/40 (35%) patients with sterile blood cultures. Among the cases with adjustment of AT, 6/23 (26%) developed RC in the further course, with no significant difference in 67/160 (42%, *p* = 0.2) cases without escalation of antibiotics. During inpatient treatment, two patients died from tumor progression and one from cholangiosepsis, while patients were successfully discharged in 180/183 cases (98%). Importantly, a short AT was not a risk factor for RC. Moreover, a short AT did not affect the mean time until RC, which was 40 days in both the short and long AT group. Positivity for MDROs (HR = 2.2, *p* = 0.0052) was an independent risk factor for RC, while placement of a metal stent was associated with a decreased risk of recurrence (HR = 0.4, *p* = 0.0383; Table 3, Figure 2A,B). Importantly, there was no significant difference among pathogens causing RC.

### 3.5. Primary versus Secondary ERC

Since risk of MDRO colonization may vary according to previous ERCs, we further evaluated whether ERC-naïve cases were comparable to cases which underwent secondary or further ERC. In fact, 25 cases which underwent primary ERC were MDRO-positive in 4 instances (16%) and developed RC in 7 instances (28%), while 158 secondary ERC cases were MDRO-positive in 47 instances (30%, *p* = 0.2) and developed RC in 66 instances (42%).

## 4. Discussion

The optimal length of AT is of high importance for patients with CAC, since excessive exposure to antibiotics favors the development of MDROs. However, the brevity of AT might put vulnerable patients at risk of reinfection. To date, the duration of AT in patients with CAC is based on clinical expertise, and concise guideline recommendations for this specific situation are urgently warranted. In this study, we address this unmet need by evaluating the impact of short AT in comparison to long AT on the recurrence rate in patients with CAC.

The current study included 124 patients with 183 cases of cholangitis, of which 73 cases (44/124 patients, 35%) experienced at least one episode of RC. There were no apparent differences between both treatment groups regarding the etiology of stenosis. Bilirubin, INR and aspartate-aminotransferase were significantly higher in the long AT group, suggesting that these patients were suffering from CAC with a higher degree of cholestasis and impairment of liver function, therefore requiring a longer course of AT. In light of our observations, short AT seems to be possible for selected patients with CAC. This is in line with a retrospective study by Van Lent et al. including 80 patients with successful drainage, in whom even three days of therapy were possible [12]. Notably, this study is the only one mentioned in the TG18, which includes a relevant part (38/80, 48%) of malignant strictures [3]. All other trials mentioned in the TG18 assessed patients with mostly benign biliary strictures and small patient numbers (*n* = 18–91), highlighting the novelty of the data of the present study. The largest recent review on this topic was published in 2021 by Haal et al. including eight studies with 938 patients [13]. The authors concluded that therapy of less than four days seems to be sufficient in patients with cholangitis and biliary stones, though the evidence is not high quality as data from randomized controlled trials are missing. No conclusion on CAC was possible due to a limited number of cases, since comparability of the management of CAC and patients with benign biliary strictures or biliary stones is highly limited. This was emphasized by a recent study by Sokal et al., who found that antibiotic resistance, inadequate empirical antibiotic therapy and mortality at day 28 were significantly more frequent in patients with CAC compared to patients with cholangitis without underlying malignancy [14]. In line with these findings, patients at our center developing cholangitis after liver transplantation may also undergo short antibiotic therapy of six or less days [7].

In the multivariate regression analysis, insertion of a metal stent was found to be independently associated with a lower risk of RC (Figure 2B). This is in line with the current recommendations from the European Society of Gastrointestinal Endoscopy and emphasizes the superiority of metal stents vs. plastic stents in this setting [15]. On the other hand, positivity for any MDRO was associated with a higher risk of RC, while MDRGN and VRE alone were not significant due to the limited number of cases in each of these groups (Figure 2A). Colonization and infections with MDROs are a frequent clinical challenge in patients with malignancies and these data highlight the urgent need for scheduled MDRO screening to avoid inadequate empirical antibiotic therapy [4,5,16]. Moreover, they emphasize the importance of optimizing the length of AT in this setting to counteract antibiotic selection pressure.

The main limitation of the current study is the lack of a standardized protocol and the retrospective single-center design. Therefore, no generalized treatment recommendation especially regarding the type of antibiotics can be made based upon our data. Furthermore, the duration of AT was chosen based on the expertise of the treating physicians. However, due to the heterogeneous and often severely impaired population, a prospective study design might be hindered and therefore retrospective data are of high value. It needs to be mentioned that this study included only non-septic patients with mild or intermediate cholangitis, while patients with grade 3 cholangitis were excluded. Likewise, only patients after ERC were included; thus, no conclusion can be drawn on patients with CAC without endoscopic intervention.

In conclusion, the current study provides some encouraging insight by demonstrating that short AT of less than seven days might be feasible for patients with mild to intermediate CAC with adequate coverage of the causative bacteria. Insertion of a metal stent should be considered if possible and frequent screening for MDROs should be recommended to optimize clinical management in this population.

## Figures and Tables

**Table 1:** Clinical characteristics of the 124 patients at baseline. Categorical variables are displayed in absolute numbers (percentages), while numerical variables are displayed as means ± standard deviation.

General Population	
Age at inclusion (years)	67.5 ± 11.7
Male sex	85 (69%)
Bile duct stent already in place	31 (25%)
**Etiology of malignant disease**	
Cholangiocarcinoma (CCA)	60 (48%)
Thereof intrahepatic CCA	17 (14%)
Thereof perihilar CCA	25 (20%)
Thereof distal CCA	9 (7%)
Thereof gallbladder carcinoma	9 (7%)
Hepatocellular carcinoma	10 (8%)
Pancreatic cancer	20 (16%
Hepatic metastases	28 (23%)
Other	6 (5%)
**Multidrug resistant bacteria (MDRO)**	
Vancomycin-resistant *Enterococci* (VRE)	20 (16%)
Multidrug-resistant Gram-negative bacteria (MDRGN)	20 (16%)
Thereof *E. coli*	14 (11%)
Thereof *K. pneumoniae*	6 (5%)
Methicillin-resistant *S. aureus*	1 (1%)
Any MDRO	31 (25%)

**Table 2:** Laboratory, endoscopic and microbiological findings of cases, stratified by duration of antibiotic therapy.

Cholangitis Episodes in 124 Patients				
	All (*n* = 183)	SAT ≤ 6 d (*n* = 74)	LAT ≥ d 7 (*n* = 109)	*p*-Value
Age, mean ± SD	67.2 ± 13	64.49 ± 15.6	68.6 ± 11.4	>0.2
Sex, male, *n* (%)	88 (71)	29 (70.7)	59 (71.1)	>0.2
Severity, *n* (%)				
Tokyo Grade I	17 (9.3)	8 (10.8)	9 (8.3)	>0.2
Tokyo Grade II	166 (90.7)	66 (89.2)	100 (91.7)	>0.2
Body temperature (°C)	37.4 ± 1.1	37.2 ± 1	37.5 ± 1.2	0.107
Leucocytes/ml	10.4 ± 4.9	9.8 ± 4.7	10.8 ± 5.1	0.168
Thrombocytes/µl	267 ± 132	265 ± 144	269 ± 125	>0.2
CRP (mg/dL; ULN = 0.5)	10 ± 7.5	8.4 ± 6	11.1 ± 8.2	0.051
Creatinine (mg/dL)	1.03 ± 0.74	1.00 ± 0.54	1.06 ± 0.85	>0.2
INR	1.22 ± 0.17	1.17 ± 0.14	1.25 ± 0.19	0.008
Total bilirubin (mg/dL)	6.2 ± 6.4	4.6 ± 5.1	7.4 ± 7	0.002
Albumin (g/dL)	3.2 ± 0.8	3.3 ± 0.6	3.2 ± 0.9	>0.2
Alkaline phosphatase (U/L)	547 ± 353	533 ± 311	557 ± 380	>0.2
gamma-Glutamyltransferase (U/L)	765 ± 572	750 ± 544	776 ± 594	>0.2
Aspartate-aminotransferase (U/L)	108 ± 73	95 ± 65	116 ± 76	0.044
Alanine-aminotransferase (U/L)	98 ± 93	92 ± 104	101 ± 84	0.062
Time from admission to ERC (h)	35.6 ± 85.4	32.9 ± 87.2	37.5 ± 84.5	0.128
ERC				
Balloon dilatation, *n* (%)	4 (2.2)	2 (2.7)	2 (1.8)	>0.2
Complete drainage, *n* (%)	134 (73.2)	57 (77)	77 (70.6)	>0.2
Papillotomy, *n* (%)	16 (8.7)	4 (5.4)	12 (11)	0.188
Stenting, *n* (%)	176 (96.2)	71 (95.6)	105 (96.3)	>0.2
Stent material (new stent)				
Plastic, *n* (%)	160 (87.4)	64 (86.5)	96 (88.1)	>0.2
Metal, *n* (%)	31 (17)	13 (17.6)	18 (16.5)	>0.2
Antibiotic duration (d, median, min-max)	7 (1–21)	6 (1–6)	9 (7–21)	
Antibiotics				
Piperacillin/Tazobactam, *n* (%)	36 (19.7)	17 (23)	19 (17.4)	>0.2
Cephalosporin, *n* (%)	76 (41.5)	33 (44.6)	43 (39.4)	>0.2
Carbapenem, *n* (%)	31 (16.9)	8 (10.8)	23 (21.1)	0.069
Fluoroquinolone, *n* (%)	43 (23.5)	16 (21.6)	28 (25.7)	>0.2
Metronidazole, *n* (%)	22 (12)	1 (1.3)	21 (19.3)	<0.001
Others†, *n* (%)	17 (9.3)	6 (8.11)	11 (10.1)	>0.2
Multidrug-resistant organisms (MDRO)	51 (27.9)	26 (35.1)	25 (22.9)	0.1
MDRGN	35 (19.1)	17 (23)	18 (16.5)	0.4
MRSA	4 (2.2)	3 (4)	1 (0.9)	0.4
VRE	31 (16.9)	17 (23)	14 (12.8)	0.1

Abbreviations: SAT = short antibiotic therapy; LAT = long antibiotic therapy; MDRGN = multidrug-resistant Gram-negative bacteria; VRE = vancomycin-resistant *Enterococci*, MRSA = methicillin-resistant *Staphylococcus aureus*. Others† comprise vancomycin, teicoplanin, tigecycline and linezolid.

**Table 3:** Multivariate regression analysis of baseline characteristics as risk factors for recurrent cholangitis.

Multivariate Regression Analysis of Risk Factors for Recurrent Cholangitis			
	Hazard Ratio	95%-Confidence Interval	*p*-Value
Antibiotic therapy (duration/d)	1.0876	0.98–1.20	0.103
Bilirubin (mg/dL)	0.9582	0.91–1.01	0.142
Colonization with any MDRO (y/n)	2.2054	1.27–3.84	0.005
Metal stent (y/n)	0.4021	0.17–0.96	0.038
Number of stents (n)	1.2015	0.90–1.61	>0.2
Short antibiotic therapy (y/n)	0.6570	0.34–1.27	>0.2
Time to ERC (h)	0.9952	0.99–1.00	0.141

Only risk factors with significance in univariate analysis are included. Abbreviations: MDRO = multidrug resistant organism, MDRGN = multidrug-resistant Gram-negative bacteria.

## Data Availability

All data can be obtained from the authors.

## Supplementary Materials

The following supporting information can be downloaded at: https://www.mdpi.com/article/10.3390/jcm12216716/s1, Table S1: Case-based analysis of multidrug resistant organism (MDRO) colonization with first and recurrent episodes of cholangitis.

## References

[1] Bowlus R., Olson K., Gershwin M. (2016). Evaluation of indeterminate biliary strictures. Nat. Rev. Gastroenterol. Hepatol..

[2] Miura F., Okamoto K., Takada T., Strasberg S.M., Asbun H.J., Pitt H.A., Gomi H., Solomkin J.S., Schlossberg D., Han H.S. (2018). Tokyo Guidelines 2018: Initial Management of Acute Biliary Infection and Flowchart for Acute Cholangitis. J. Hepatobiliary Pancreat. Sci..

[3] Gomi H., Solomkin J.S., Schlossberg D., Okamoto K., Takada T., Strasberg S.M., Ukai T., Endo I., Iwashita Y., Hibi T. (2018). Tokyo Guidelines 2018: Antimicrobial Therapy for Acute Cholangitis and Cholecystitis. J. Hepatobiliary Pancreat. Sci..

[4] Himmelsbach V., Knabe M., Ferstl P.G., Peiffer K.H., Stratmann J.A., Wichelhaus T.A., Hogardt M., Kempf V.A.J., Zeuzem S., Waidmann O. (2021). Colonization with Multidrug-Resistant Organisms Impairs Survival in Patients with Hepatocellular Carcinoma. J. Cancer Res. Clin. Oncol..

[5] Stratmann J.A., Lacko R., Ballo O., Shaid S., Gleiber W., Vehreschild M.J.G.T., Wichelhaus T., Reinheimer C., Göttig S., Kempf V.A.J. (2020). Colonization with Multi-Drug-Resistant Organisms Negatively Impacts Survival in Patients with Non-Small Cell Lung Cancer. PLoS ONE.

[6] Perdikouri E.I.A., Arvaniti K., Lathyris D., Kiouti F.A., Siskou E., Haidich A.B., Papandreou C. (2019). Infections Due to Multidrug-Resistant Bacteria in Oncological Patients: Insights from a Five-Year Epidemiological and Clinical Analysis. Microorganisms.

[7] Ferstl P.G., Queck A., Bremer K., Filmann N., Weiler N., Welker M., Waidmann O., Knabe M., Bechstein W.O., Hogardt M. (2022). Comparison of Short-course Antibiotic Therapy of Six or Less Days with a Longer Treatment in Patients with Cholangitis after Liver Transplantation. Transpl. Infect. Dis..

[8] Magiorakos A.P., Srinivasan A., Carey R.B., Carmeli Y., Falagas M.E., Giske C.G., Harbarth S., Hindler J.F., Kahlmeter G., Olsson-Liljequist B. (2012). Multidrug-Resistant, Extensively Drug-Resistant and Pandrug-Resistant Bacteria: An International Expert Proposal for Interim Standard Definitions for Acquired Resistance. Clin. Microbiol. Infect..

[9] Geffers C., Gastmeier P. (2011). Nosokomiale Infektionen Und Multiresistente Erreger in Deutschland: Epidemiologische Daten Aus Dem Krankenhaus-Infektions-Surveillance-System. Dtsch. Arztebl..

[10] Ferstl P.G., Filmann N., Brandt C., Zeuzem S., Hogardt M., Kempf V.A.J., Müller M., Waidmann O., Reinheimer C. (2017). The Impact of Carbapenem Resistance on Clinical Deterioration and Mortality in Patients with Liver Disease. Liver Int..

[11] Reinheimer C., Kempf V., Göttig S., Hogardt M., Wichelhaus T.A., O’Rourke F., Brandt C. (2016). Multidrug-resistant organisms detected in refugee patients admitted to a University Hospital, Germany June–December 2015. Eurosurveillance.

[12] Van Lent A.U.G., Bartelsman J.F.W.M., Tytgat G.N.J., Speelman P., Prins J.M. (2002). Duration of Antibiotic Therapy for Cholangitis after Successful Endoscopic Drainage of the Biliary Tract. Gastrointest. Endosc..

[13] Haal S., Wielenga M.C.B., Fockens P., Leseman C.A., Ponsioen C.Y., van Soest E.J., van Wanrooij R.L.J., Sieswerda E., Voermans R.P. (2021). Antibiotic Therapy of 3 Days May Be Sufficient After Biliary Drainage for Acute Cholangitis: A Systematic Review. Dig. Dis. Sci..

[14] Sokal A., Chawki S., Nguyen Y., Sauvanet A., Ponsot P., Maire F., Fantin B., de Lastours V. (2022). Specificities of Acute Cholangitis in Patients with Cancer: A Retrospective Comparative Study of 130 Episodes. Eur. J. Clin. Microbiol. Infect. Dis..

[15] Dumonceau J.M., Tringali A., Papanikolaou I.S., Blero D., Mangiavillano B., Schmidt A., Vanbiervliet G., Costamagna G., Devière J., García-Cano J. (2018). Endoscopic Biliary Stenting: Indications, Choice of Stents, and Results: European Society of Gastrointestinal Endoscopy (ESGE) Clinical Guideline–Updated October 2017. Endoscopy.

[16] Ballo O., Tarazzit I., Stratmann J., Reinheimer C., Hogardt M., Wichelhaus T.A., Kempf V., Serve H., Finkelmeier F., Brandts C. (2019). Colonization with Multidrug Resistant Organisms Determines the Clinical Course of Patients with Acute Myeloid Leukemia Undergoing Intensive Induction Chemotherapy. PLoS ONE.

