# Supplementary material for: Short Versus Long Antibiotic Therapy and Risk of Recurrence of Acute Cholangitis Due to Malignant Biliary Strictures
Source: J Clin Med. 2023 Oct 24;12(21):6716. doi: 10.3390/jcm12216716 (PMC10648096; doi:10.3390/jcm12216716)
Supplement: Supplementary file 1 [file jcm-12-06716-s001.zip › jcm-2620973-supplementary.pdf]

Table S1. Case-based analysis of multidrug resistant organism (MDRO) colonization with first and recurrent episodes of cholangitis.

	All cases (*n* = 183)	RC within 28d (*n* = 14)	Any RC (*n* = 73)
No MDRO	132 (72.1%)	8 (57.1%)	46 (63%)
MDRGN	35 (19.1%)	5 (35.7%)	20 (27.4%)
VRE	31 (16.9%)	2 (14.3%)	15 (20.6%)
MDRGN + VRE	15 (8.2%)	1 (7.1%)	8 (11%)
MDRGN or VRE	36 (19.7%)	5 (35.7%)	19 (26%)
MDRGN or VRE or MRSA†	32 (17.5%)	5 (35.7%)	16 (21.9%)

Abbreviations: RC = Recurrent cholangitis. MDRGN = Multidrug-resistant Gram-negative bacteria. MRSA = Methicillin-resistant *Staphylococcus aureus*. VRE = Vancomycin-resistant *Enterococci.* †All four Patients with MRSA were also positive for MDRGN.
